# Cardiovascular and autonomic modulation during nighttime rest under real-world conditions in miners exposed to chronic intermittent hypoxia

**DOI:** 10.3389/fphys.2026.1747092

**Published:** 2026-03-30

**Authors:** Morin Lang, José Luis Vega, Grzegorz Bilo, Edgardo Opazo-Diaz, Gonzalo Araya Diaz, Martina Anna Maggioni

**Affiliations:** 1 Departamento de Kinesiología, Facultad de Medicina, Universidad de Chile, Santiago, Chile; 2 Facultad de Ciencias Biológicas, Departamento de Fisiología, Universidad de Concepción, Concepción, Chile; 3 Department of Medicine and Surgery, University of Milano-Bicocca, Milan, Italy; 4 Department of Cardiology, Istituto Auxologico Italiano, Istituto di Ricerca e Cura a Carattere Scientifico (IRCCS), Milan, Italy; 5 Compañía Minera Doña Inés de Collahuasi, Iquique, Chile; 6 Charité-Universitätsmedizin Berlin, Charité Center for Global Health and Center for Space Medicine and Extreme Environments Berlin, Berlin, Germany; 7 Heidelberg Institute of Global Health, University Hospital, Heidelberg University, Heidelberg, Germany; 8 Department of Biomedical Sciences for Health, Università degli Studi di Milano, Milano, Italy

**Keywords:** ambulatory blood pressure, chronic intermittent hypoxia, heart rate variability, high altitude, nocturnal blood pressure

## Abstract

**Background:**

Chilean mine workers commuting between sea level (SL) and high altitude (HA) are repeatedly exposed to chronic intermittent hypoxia (CIH). However, cardiovascular responses during nighttime rest, particularly the combined behavior of blood pressure (BP) and heart rate variability (HRV), remain insufficiently characterized under real-world occupational field conditions.

**Methods:**

Nineteen male mine workers (35–55 years) with over 10 years of rotational work (7-on/7-off shifts) were evaluated at SL (<500 m) and HA (∼3,800 m). Participants were classified as normotensive (NT, n = 9) or hypertensive (HT, n = 10). Resting SpO_2_, continuous 1-lead ECG and ambulatory BP monitoring were recorded simultaneously during a standardized 4-h nocturnal window (00:30–04:30 h), selected based on sustained absence of movement assessed by accelerometry. HRV was analyzed using time-, frequency-, and non-linear indices.

**Results:**

SpO_2_ significantly decreased from SL to HA (97.6% ± 0.5% vs. 87.8% ± 3.0%; p < 0.001), with a greater decline in hypertensive workers (p < 0.001). HA exposure significantly increased systolic and diastolic BP during the analyzed nighttime window (p < 0.05) and mean heart rate (p < 0.05) in both groups, without a significant group × altitude interaction. Parasympathetic-related HRV indices (RMSSD and SampEn) decreased significantly at HA, while SDNN increased under CIH exposure. Correlations between HRV and nocturnal BP were weak and non-significant in the overall sample.

**Conclusion:**

CIH exposure is associated with reduced oxygen saturation, elevated BP, and decreased vagal modulation during a standardized nighttime rest period under real-world conditions. Although HRV and BP changed in parallel at the group level, their individual associations were weak, suggesting that short-term cardiac vagal modulation alone does not fully explain BP responses during this predefined rest window under hypoxic stress. These findings describe field-based autonomic–hemodynamic responses during nighttime rest in CIH-exposed male workers.

## Introduction

Chilean mine workers working in 7-on/7-off shifts at altitudes above 3,000 m face unique physiological challenges. This rotating work system, alternating between high-altitude (HA) exposure and off-shift periods at low altitude or sea level (SL), constitutes a real-world occupational model of chronic intermittent hypobaric hypoxia (CIH) (Ministerio de Salud de Chile, 2013). Although mining has historically been considered physically demanding, modern large-scale operations often involve automated systems, supervisory tasks, prolonged sitting, and shift-based control-room work, resulting in heterogeneous physical workloads across roles. This occupational and environmental pattern has been associated with increased body mass index, reduced physical activity, poor sleep quality, and higher BP responses at altitude, particularly in hypertensive workers, suggesting impaired autonomic regulation (Pizarro-Montaner et al., 2020; Lang et al., 2021). Long-term CIH, as experienced by miners and military personnel, has been linked to increased cardiovascular morbidity and structural cardiac alterations (Aragón-Vela et al., 2020; González-Candia et al., 2023; Pedreros-Lobos et al., 2021).

CIH generates repeated hypoxia–reoxygenation cycles that impose significant cardiovascular strain. Arterial oxygen saturation (SpO_2_) serves as a practical field-based indicator of hypoxic severity and reflects the magnitude of the hypobaric stimulus in CIH-exposed workers (Ministerio de Salud de Chile, 2013; Pizarro-Montaner et al., 2020; Lang et al., 2021; Aragón-Vela et al., 2020; González-Candia et al., 2023; Pedreros-Lobos et al., 2021). Reductions in SpO_2_ at altitude have been consistently associated with autonomic imbalance, sympathetic activation, and altered baroreflex control (Halliwill and Minson, 2002; Parati et al., 2014a; Bilo et al., 2015). In addition to SpO_2_, hematological adaptations such as increased hemoglobin concentration and hematocrit reflect chronic acclimatization to sustained hypobaric hypoxia and may modulate cardiovascular responses under CIH conditions (Heinicke et al., 2003). Hypobaric hypoxia triggers multiple mechanisms contributing to BP elevation, including heightened peripheral chemoreflex sensitivity, sympathetic activation, ventilatory instability with periodic breathing during nocturnal rest, and baroreflex resetting toward higher operating pressures (Halliwill and Minson, 2002; Parati et al., 2014a; Bilo et al., 2015; Heinicke et al., 2003; Lombardi et al., 2013; Torlasco et al., 2020; Ainslie et al., 2007). Additional contributors, such as endothelial dysfunction, arterial stiffness, altered renin–angiotensin–aldosterone activity, and hypoxia-inducible pathways, may further modulate vascular responses under CIH (Bourdillon et al., 2019; Yanamandra et al., 2017; Revera et al., 2017).

Heart rate variability (HRV) is a validated non-invasive tool to assess cardiac autonomic modulation and carries prognostic value across cardiovascular conditions such as hypertension, diabetes, and heart failure (Parati et al., 1995; Hsu et al., 2015; Yılmaz et al., 2021; de Oliveira et al., 2020; Thayer et al., 2010; Huikuri and Stein, 2013). HRV assessed during nocturnal rest periods is particularly informative, as sleep is generally associated with enhanced parasympathetic-related cardiac modulation, physiological BP dipping, and autonomic recovery (Chouchou and Desseilles, 2014; Stein and Pu, 2012; Trinder et al., 2001). However, HRV indices, particularly HF power and non-linear measures, may vary according to sleep stage composition. In field-based settings without polysomnographic staging, HRV reflects overall cardiac autonomic modulation during the analyzed rest period rather than stage-specific autonomic dynamics. Vagally mediated indices, such as RMSSD and non-linear short-term metrics, are considered sensitive markers of parasympathetic-related cardiac modulation during nocturnal recordings (Shaffer and Ginsberg, 2017; Laborde et al., 2017). Under hypoxic stress, however, CIH has been associated with reduced vagal modulation, increased sympathetic activity, impaired baroreflex sensitivity, and promote oxidative stress and carotid body hyperexcitability (Tamisier et al., 2011; Del Rio et al., 2010; Castro-Grattoni et al., 2016; Iturriaga et al., 2014), mechanisms that may contribute to nocturnal BP elevation (Parati et al., 2014a; Bilo et al., 2015; Heinicke et al., 2003; Lombardi et al., 2013; Torlasco et al., 2020).

Mine workers chronically exposed to CIH show reduced HRV, increased 24-h and nighttime BP, and blunted dipping patterns, particularly among those with hypertension (Lang et al., 2021; Lang et al., 2022; Richalet et al., 2002; Farias et al., 2013; Moraga et al., 2014). Despite these findings, few studies have simultaneously characterized BP and HRV during nighttime rest using synchronized, field-based measurements under real-world occupational conditions, and detailed comparisons between normotensive and hypertensive mine workers remain scarce. Most available evidence derives from laboratory-based protocols or from studies focusing on single autonomic markers rather than integrated hemodynamic–autonomic responses. In this context, analyzing a standardized nighttime rest window allows temporal alignment between ambulatory BP measurements and HRV indices under field conditions, providing insight into short-term autonomic–hemodynamic patterns. As nighttime BP and vagal-related HRV indices are established predictors of cardiovascular risk in broader populations (Tsuji et al., 1994; Dekker et al., 1997), understanding cardiovascular responses during nighttime rest under CIH is clinically relevant.

In this occupational model, chronic intermittent hypoxia results from repeated exposure to sustained hypobaric hypoxia during shift-based high-altitude work, which differs fundamentally from short-cycle intermittent hypoxia models.

The aim of this study was to characterize the integrated patterns of BP and HRV during a standardized nighttime rest period in mine workers exposed to CIH and to examine their association according to blood pressure status.

## Methods

### Participants

A total of 19 male mine workers (mean age 47 ± 7 years; BMI 28.8 ± 2.6 kg/m^2^) were initially enrolled in this observational, cross-sectional field-based sub-study. All participants worked in a rotational shift system (7 days-on at HA >3,800–4,200 m above SL, followed by 7 days-off at SL < 500 m above SL).

Participants were mine workers employed at an open-pit mine located in northern Chile, operating under open-air high-altitude conditions, and were selected from a cohort previously described by Lang et al. (2021). All participants worked under a rotational shift system consisting of 7 consecutive days at work followed by 7 days off. Work shifts alternated between day and night across rotations; however, all physiological assessments were conducted during the daytime work rotation, ensuring homogeneous measurement conditions. Smoking status and sleep apnea history were recorded as part of the clinical assessment.

Eligibility criteria included: age 19–60 years, BMI <35 kg/m^2^, permanent residence below 500 m altitude, no hypertension history (i.e., no pharmacological treatment for hypertension, NT group) or presence of hypertension, but on treatment (HT group). Participants with cardiovascular diseases other than hypertension, or with suspected or confirmed secondary hypertension, diabetes mellitus were excluded. No changes in medication occurred between SL and HA assessments. Participants on beta-blockers were excluded due to interference with HRV interpretation.

For the present study, focused on nocturnal cardiovascular and autonomic behavior during rest, a subsample of 19 participants (9 NT and 10 HT) was selected based on achieving ≥4 h of artifact-free ECG data during a standardized nocturnal recording window. ECG quality was assessed after initial offline inspection of ECG waveforms. In addition, data from the tri-axial accelerometer integrated into the ECG device were examined to confirm sustained absence of gross body movement during the analyzed period, which was used as a behavioral proxy of nocturnal rest, independent of sleep stage.

Hemoglobin and hematocrit values were obtained from routine occupational health examinations performed approximately 1 year prior to the study. These records are part of mandatory medical surveillance programs for workers exposed to CIH. All participants met national occupational criteria for high-altitude work according to the Chilean Ministry of Health Technical Guidelines on CIH (Ministerio de Salud de Chile, 2013). No participant presented anemia or hemoglobin values exceeding contraindication thresholds for high-altitude work. Given that these measurements were not obtained simultaneously with physiological recordings, they were used exclusively for descriptive and contextual characterization and not included in statistical analyses.

All participants were employees of a large Chilean mining company for at least 2 years. The majority were equipment operators (60%), followed by administrative staff (30%) and maintenance workers (10%). Although occupational roles varied, all participants were evaluated during a standardized nighttime rest period, independent of active work tasks, thereby minimizing the immediate influence of daytime workload on the analyzed autonomic indices. All participants provided written informed consent. The study protocol was approved by the Ethics Committee of the University of Antofagasta (approval no. 181/2019) and conducted in accordance with the Declaration of Helsinki (2013).

### Experimental procedure

**FIGURE 1:**
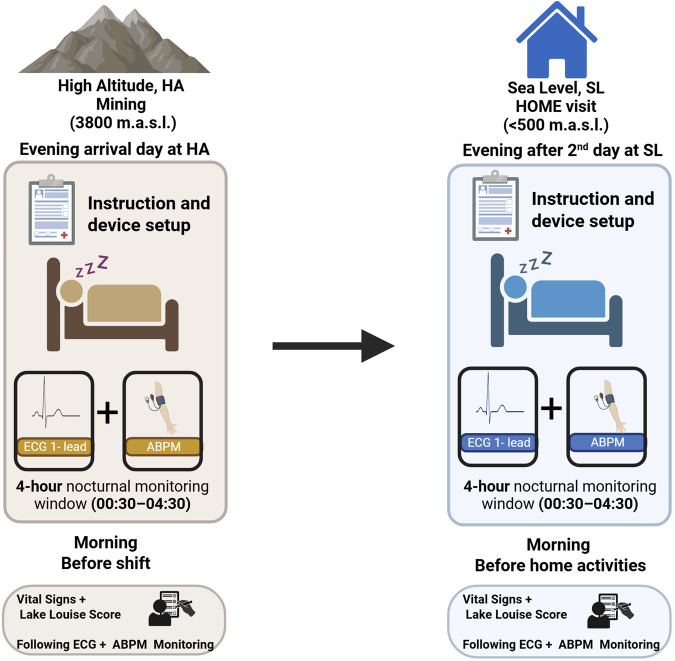
Study design and measurement timeline at high altitude (HA) and sea level (SL). Participants underwent assessments during two separate exposure periods: the first night after arrival at high altitude (mining facility at ∼3,800 m a.s.l.) and after the second day at sea level (<500 m a.s.l., home visit). On both occasions, devices were set up in the evening for overnight monitoring. A 4-h nocturnal monitoring window (00:30–04:30 a.m.) was used for HRV and BP data acquisition, derived from continuous 1-lead ECG and ambulatory blood pressure monitoring (ABPM). Vital signs and Lake Louise Score were assessed the following morning, prior to shift start (at HA) or before daily activities (at SL). ECG, electrocardiogram; ABPM, ambulatory blood pressure monitoring; HRV, heart rate variability; HA, high altitude; SL, sea level.

The study design and measurement timeline are presented in Figure 1. Each participant completed two 36-h monitoring sessions following previously published protocols (Lang et al., 2021; Lang et al., 2022):

1. High altitude (HA): first night after arrival to the mining facility (∼3,800 m a.s.l.) at the start of the 7-on work shift.
2. Sea level (SL): second or third day of the off-shift period (<500 m).

All participants were assessed first at high altitude and subsequently at sea level. This first-night recording reflects the onset of the hypoxic phase within a long-standing rotational chronic intermittent hypoxia (CIH) model, rather than primary acute altitude exposure in unacclimatized individuals.

Randomization of the testing order was considered but was not feasible due to logistical constraints related to the fixed rotational work system (7 days-on/7 days-off) and the need to perform assessments during active work periods at high altitude. Given the long-standing exposure to chronic intermittent hypoxia, potential order effects were considered unlikely to fully account for the observed altitude-related differences. Years of high-altitude exposure were therefore included as a descriptive variable.

Participants simultaneously wore a miniaturized 1-lead ECG device and a validated ambulatory blood pressure monitor (ABPM). Continuous ECG signals were recorded using a single-lead device (Faros 180, Bittium, Finland) at a sampling frequency of 250 Hz. The device integrates a tri-axial accelerometer, allowing concurrent assessment of body movement during recording. The Faros system has been widely used in ambulatory HRV research and meets established methodological standards for time- and frequency-domain HRV analysis. ECG electrodes were positioned on the upper right chest and left anterior axillary line according to manufacturer guidelines.

Twenty-four-hour ABPM (TM-2430, AND, Japan) was applied to the non-dominant arm. Measurements were programmed every 20 min during daytime (07:00–22:00) and every 30 min during nighttime (22:00–07:00). Recordings with ≥70% valid readings were included according to predefined quality criteria. Participants were instructed to maintain usual daily activities while avoiding strenuous exercise and to remain still during BP measurements.

ECG monitoring lasted 36 h with minimal interruption. A standardized nighttime rest window (00:30–04:30 h) was extracted for HRV and nocturnal BP analyses. To increase the likelihood that this interval corresponded to nocturnal rest, accelerometry data were examined and segments characterized by sustained absence of body movement were selected. This approach served as a behavioral proxy for nocturnal rest, independent of sleep-stage assessment, and is consistent with field-based monitoring strategies where polysomnography is not feasible.

The standardized nighttime rest window (00:30–04:30 h) was predefined prior to data analysis to ensure temporal consistency and comparability across participants and altitude conditions. Only recordings containing ≥4 consecutive hours within this window were included in HRV analyses. Recordings outside this predefined interval were not analyzed to minimize variability related to early awakening, pre-shift mobilization, and morning sympathetic activation.

### Clinical measurements and ambulatory blood pressure monitoring

Baseline anthropometry measurements, peripheral oxygen saturation (SpO_2_), and heart rate (HR) were collected following standardized procedures (Lang et al., 2022).

Hypertension was defined based on previous medical diagnosis and current antihypertensive treatment. Antihypertensive therapy consisted of standard first-line drug classes, including angiotensin receptor blockers, angiotensin-converting enzyme inhibitors, calcium channel blockers, beta-blockers, and thiazide diuretics, either as monotherapy or in combination. The pharmacological profile and available medical records were consistent with primary (essential) hypertension, and no participant had a documented secondary cause of hypertension.

Conventional BP was obtained with validated oscillometric devices (UA-767Plus, AND, Japan). SpO_2_ and HR were measured using a pulse oximeter (Vantage 9590, NONIN, United States). Acute Mountain Sickness (AMS) was diagnosed based on the Lake Louise Score System (LLS) and defined as the presence of headache with total LLS score of three or more points from the four rated symptoms (headache, gastrointestinal symptoms, fatigue and/or weakness and dizziness). AMS was then classified as mild (3-5 points), moderate (6-9 points) or severe (9–12 points) (Roach et al., 2018). Body height and weight were measured with a validated scale and an altimeter and waist circumference with a meter tape.

Daytime and nocturnal BP and HR values were averaged based on predefined clock-time intervals, independent of sleep-stage information.

During the standardized nighttime rest window (00:30–04:30 h), eight BP measurements were available per participant under both conditions. Mean nocturnal systolic and diastolic BP values were calculated as the arithmetic average of these eight readings for each condition.

Nocturnal BP fall (dipping) was calculated as:

$$\text{Dipping }\left(\%\right)=[\left(\text{mean daytime SBP }-\text{ mean nocturnal SBP}\right)/\text{ mean daytime SBP}]\times 100$$

A nocturnal SBP decline ≥10% was classified as normal dipping, whereas <10% was classified as non-dipping, in accordance with established ABPM guidelines (Parati et al., 2014b; Parati et al., 2025).

### ECG acquisition and heart rate variability analysis

Continuous 1-lead ECG signals were recorded at 250 Hz and synchronized with ambulatory blood pressure monitoring (ABPM). For HRV analysis, the standardized 4-h nocturnal window described above was extracted for each participant and altitude condition.

ECG preprocessing included baseline correction, automatic detection of normal-to-normal (NN) intervals, and artifact correction according to established international recommendations (Task Force of the European Society of Cardiology and the North American Society of Pacing and Electrophysiology, 1996). All analyses were performed using Kubios HRV Premium software (version 3.5.0, Kubios Ltd., Kuopio, Finland) (Tarvainen et al., 2014).

RR interval series were interpolated at 4 Hz prior to spectral analysis. Frequency-domain indices were computed using Fast Fourier Transform (FFT)-based power spectral analysis with 300-s segments and 50% overlap. Trend removal was applied using the smoothness priors method, and automatic artifact correction was implemented. Identical preprocessing and analysis procedures were applied to both sea level and high-altitude recordings.

The low-frequency (LF) band was defined as 0.04–0.15 Hz and the high-frequency (HF) band as 0.15–0.40 Hz. Spectral power was expressed in absolute units (ms^2^) and normalized units (n.u.) when appropriate.

Time-domain, frequency-domain, and non-linear indices were calculated in accordance with current methodological standards (Task Force of the European Society of Cardiology and the North American Society of Pacing and Electrophysiology, 1996; Tarvainen et al., 2014; Goldstein et al., 2011; Castiglioni et al., 2025). RMSSD was used as the primary short-term marker of cardiac parasympathetic modulation. SDNN and the LF/HF ratio were included for descriptive purposes only, given their limited specificity in short nocturnal segments. Sample entropy (SampEn; m = 2, r = 0.2) was calculated as an index of autonomic complexity.

To account for potential respiratory influences on HRV, respiratory rate was estimated using ECG-derived respiration (EDR, breaths·min^-1^) within the analyzed nocturnal window. EDR values were compared between altitude conditions to evaluate potential respiratory confounding effects.

### Statistical analysis

Data distribution was assessed for normality using the Shapiro–Wilk test, and homogeneity of variances was evaluated using Levene’s test. As most variables did not meet the assumptions of normality and/or homogeneity, non-parametric statistical methods were applied throughout the analysis.

Paired comparisons between sea level and high altitude were performed using the Wilcoxon matched-pairs signed-rank test. Between-group comparisons (normotensive vs. hypertensive participants) were conducted using the Mann–Whitney U test. Effect sizes for non-parametric comparisons were calculated as r = Z/√n and interpreted according to conventional thresholds.

Correlation analyses were performed using Spearman’s rank correlation coefficient due to non-normal data distribution. For correlation analyses, exact two-tailed p-values and sample sizes (n) are reported.

Descriptive data are presented as mean ± standard deviation (SD). All statistical tests were two-tailed, and a p value <0.05 was considered statistically significant.

Given the repeated-measures design (sea level vs. high altitude), paired non-parametric tests were applied to account for within-subject differences between conditions. Due to the limited sample size and absence of additional hierarchical levels, mixed-effects modeling was not pursued.

Analyses were performed using GraphPad Prism 4.50.3 (GraphPad Software, San Diego, CA, United States).

## Results

### Participant characteristics

Baseline demographic, anthropometric, and clinical characteristics at sea level are summarized in Table 1. The study population included 10 hypertensive and 9 normotensive participants. Seven participants (36.8%) were current smokers, and four participants (21.1%) reported a history compatible with sleep apnea. The distribution of smoking status and sleep apnea history according to blood pressure status is detailed in Table 1. Historical occupational hemoglobin values ranged between 15.2 and 18.9 g/dL, all within occupational nationally accepted hematological ranges for high-altitude work.

**TABLE 1:** Demographic, anthropometric, and clinical characteristics of the study population at sea level.

Characteristics	All (n = 19)	Normotensive (NT, n = 9)	Hypertensive (HT, n = 10)
Age (years)	47.0 ± 7.3	44.0 ± 5.3	52.0 ± 8.1
Body mass index (kg·m−2)	28.8 ± 2.6	28.8 ± 2.5	29.1 ± 2.9
SpO_2_ (%)	97.5 ± 0.5	97.5 ± 0.7	98.0 ± 0.2
Heart rate (bpm)	66.5 ± 15.9	62.5 ± 9.1	66.5 ± 9.3
Systolic blood pressure (mmHg)	122.0 ± 9.1	122.1 ± 8.4	118.6 ± 6.1
Diastolic blood pressure (mmHg)	71.1 ± 8.2	70.3 ± 8.5	71.4 ± 5.7
Current smokers, n (%)	7 (36.8)	4 (44.4)	3 (30.0)
Sleep apnea history, n (%)	4 (21.1)	3 (33.3)	1 (10.0)
Exposure time (years)	9.8 ± 4.4	8.3 ± 6.9	10.2 ± 2.1

Data are presented as mean ± SD (standard deviation) or as n (%), as appropriate. SpO_2_: Peripheral oxygen saturation. Smoking status and sleep apnea history were recorded as dichotomous variables. No participant received specific treatment for sleep apnea during the evaluation period.

The sample included both normotensive and hypertensive participants, with antihypertensive therapy maintained consistently across altitudes. The treatment included angiotensin II receptor blockers (ARBs) in 6 participants, calcium channel blockers (CCBs) in 1, combined ARB + CCB therapy in 2, and angiotensin-converting enzyme (ACE) inhibitors in 1 participant. No participants were treated with beta-blockers, in accordance with the study’s exclusion criteria related to HRV analysis.

### Cardiorespiratory variables and acute mountain sickness

#### Arterial oxygen saturation

Resting arterial oxygen saturation (SpO_2_) significantly decreased from sea level to high altitude in the overall sample (97.6% ± 0.5% vs. 87.8% ± 3.0%; p < 0.001; r = −0.88). The reduction was observed in both normotensive (Δ = −9.1% ± 3.0%, p < 0.001) and hypertensive participants (Δ = −10.4% ± 3.0%, p = 0.002), with a greater absolute decline in the hypertensive group (between-group comparison, p < 0.001).

#### Heart rate

Mean nocturnal heart rate (HR) increased significantly at high altitude in the overall sample (60.6 ± 7.5 vs. 79.5 ± 13.6 beats·min^-1^; p < 0.001). Both normotensive (Δ = +18.5 ± 12.4 beats·min^-1^, p = 0.039) and hypertensive participants (Δ = +19.2 ± 12.6 beats·min^-1^, p = 0.012) exhibited significant HR elevations. Between-group differences in HR change reached statistical significance (p = 0.020); however, interpretation should consider the limited sample size.

#### Acute mountain sickness

The distribution of Lake Louise Scores (LLS) and the prevalence of acute mountain sickness (AMS) are presented in Supplementary Figure S1. A greater proportion of normotensive participants met the diagnostic criteria for AMS (LLS ≥3 with headache) compared with hypertensive participants. All reported symptoms were mild and transient, and none required pharmacological treatment or descent. Although normotensive participants presented numerically higher LLS values, between-group differences were not statistically significant.

#### Nocturnal blood pressure and dipping status

**FIGURE 2:**
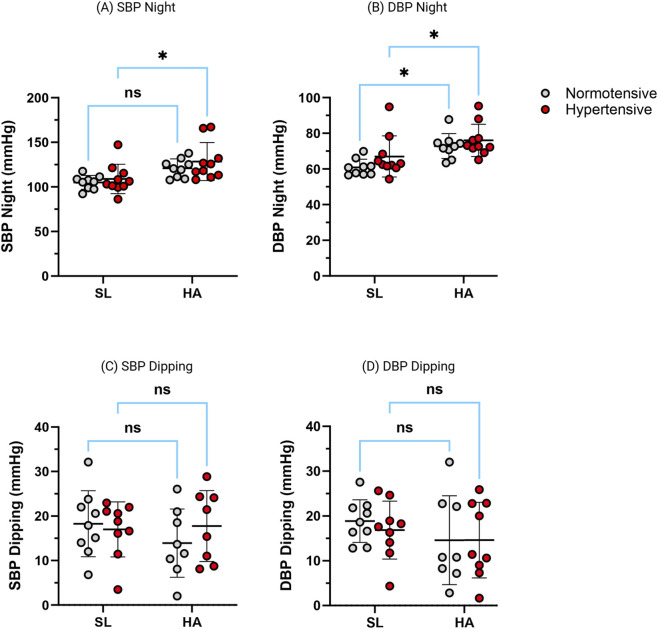
Effect of chronic intermittent hypoxia (CIH) on nocturnal systolic and diastolic blood pressure and night-time dipping. Graphs show individual values and group means ± SD for systolic blood pressure (SBP), diastolic blood pressure (DBP), and their respective night-time dipping values in normotensive (white circles) and hypertensive (red circles) participants, measured at sea level (SL) and high altitude (HA). Top row: **(A)** Nocturnal systolic blood pressure (SBP Night, mmHg); **(B)** Nocturnal diastolic blood pressure (DBP Night, mmHg). Bottom row: **(C)** SBP dipping; **(D)** DBP dipping. Asterisks indicate within-group comparisons between SL and HA (Wilcoxon matched-pairs signed-rank test). Between-group comparisons (normotensive vs. hypertensive) were performed using the Mann–Whitney U test. p < 0.05, p < 0.01, p < 0.001; ns = not significant. Abbreviations: CIH, chronic intermittent hypoxia; SL, sea level; HA, high altitude.

As illustrated in Figure 2, high-altitude exposure was associated with a significant increase in mean nocturnal systolic and diastolic blood pressure in the overall sample (p < 0.01). Within-group analyses indicated that hypertensive participants exhibited significant increases in nocturnal SBP and DBP at high altitude compared with sea level, whereas changes in normotensive participants did not reach statistical significance.

The nocturnal BP dipping percentage did not differ significantly between sea level and high altitude (p = 0.19; r = −0.45). Although a higher proportion of hypertensive participants displayed a non-dipping profile (<10% nocturnal BP fall) at high altitude, these differences were not statistically significant.

#### Heart rate variability during nighttime rest

**FIGURE 3:**
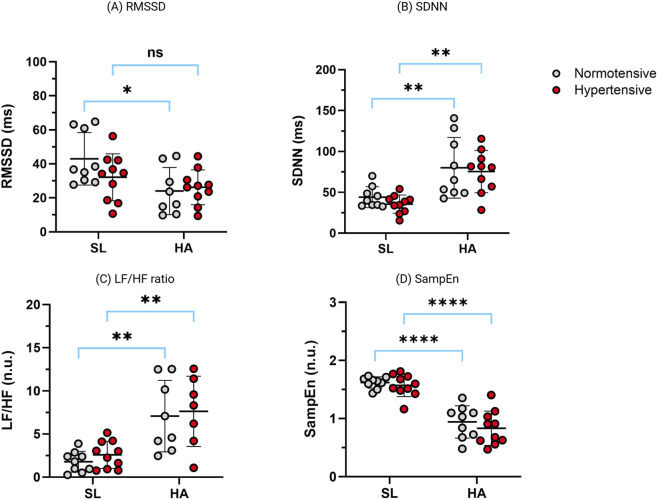
Nocturnal heart rate variability (HRV) indices in normotensive (white circles) and hypertensive (red circles) mine workers at sea level (SL) and high altitude (HA). Panels show: **(A)** RMSSD, **(B)** SDNN, **(C)** LF/HF ratio, and **(D)** sample entropy (SampEn). Individual data points and group means ± SD are displayed. Asterisks indicate within-group comparisons between SL and HA (Wilcoxon matched-pairs signed-rank test). No statistically significant between-group differences (normotensive vs. hypertensive) were observed at either altitude (Mann–Whitney U test). *p < 0.05; **p < 0.01; ***p < 0.001; ***p < 0.0001; ns = not significant. Abbreviations: RMSSD, root mean square of successive differences; SDNN, standard deviation of normal-to-normal intervals; LF/HF, low-frequency to high-frequency ratio; SL, sea level; HA, high altitude; NT, normotensive; HT, hypertensive.

Changes in nocturnal heart rate variability indices at sea level (SL) and high altitude (HA), stratified by blood pressure status, are shown in Figure 3. In both normotensive and hypertensive participants, exposure to HA was associated with a significant reduction in parasympathetic-related indices, evidenced by lower RMSSD and sample entropy (SampEn). SDNN increased significantly at HA in both groups.

Absolute HF power (ms^2^) decreased significantly at high altitude compared to sea level (Wilcoxon signed-rank test, p = 0.0005), whereas absolute LF power (ms^2^) increased (p = 0.002). These altitude-related changes were consistent across normotensive and hypertensive subgroups. No significant between-group differences were observed at either altitude (Mann–Whitney U test, all p > 0.05). Absolute spectral power values are presented in Supplementary Figure S2.

Although RMSSD values were numerically lower in hypertensive participants at both SL and HA, variability across individuals resulted in no statistically significant between-group differences. Similarly, no significant between-group differences were observed for SampEn, SDNN, or the LF/HF ratio at either altitude. Changes in the LF/HF ratio with altitude were comparable between normotensive and hypertensive participants and are therefore presented for descriptive purposes only.

No significant differences were observed in ECG-derived respiration (EDR) between sea level and high altitude in either group (see Supplementary Figure S3).

### Relationship between cardiac autonomic modulation and blood pressure

**FIGURE 4:**
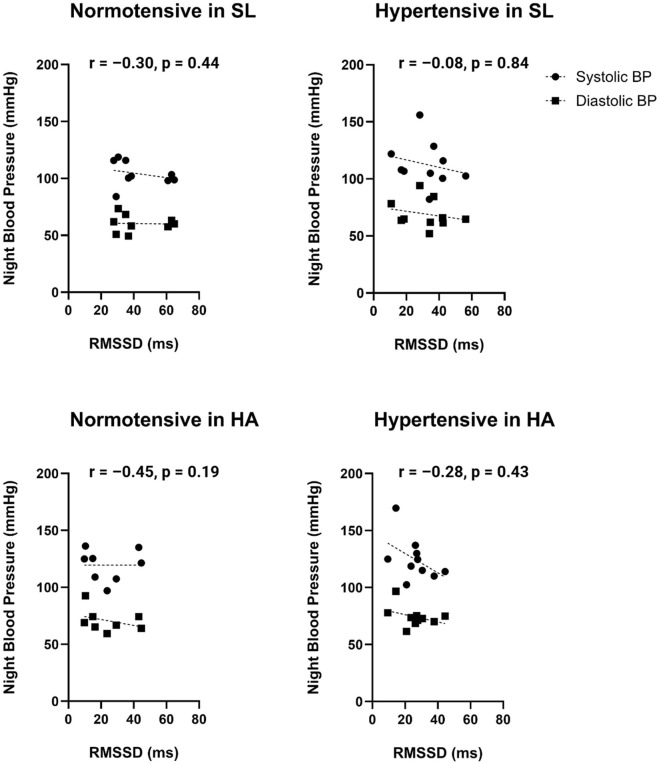
Relationship between nocturnal cardiac parasympathetic modulation and mean nocturnal blood pressure at sea level (SL) and high altitude (HA). Scatter plots illustrate the association between root mean square of successive differences (RMSSD) and mean nocturnal systolic blood pressure (SBP, circles) and diastolic blood pressure (DBP, squares) in normotensive and hypertensive participants under both altitude conditions. Each point represents one participant. Mean nocturnal BP values were calculated as the arithmetic average of eight ambulatory blood pressure measurements Spearman correlation coefficients (r) and corresponding two-tailed p-values are displayed within each panel obtained every 30 min during the standardized 4-h nocturnal window (00:30–04:30 h). Given the small subgroup sizes (n = 9–10), these correlations should be interpreted cautiously. No statistically significant associations were observed between RMSSD and nocturnal SBP or DBP at either altitude condition.

Correlation analyses between nocturnal cardiac autonomic modulation and blood pressure are shown in Figure 4. RMSSD, selected *a priori* as the primary vagal marker, showed weak and non-significant associations with nocturnal blood pressure across all subgroups. Correlation analyses were conducted separately in normotensive (n = 9) and hypertensive (n = 10) participants at each altitude.

In normotensive participants at sea level, Spearman correlations between RMSSD and nocturnal systolic BP (r_s_ = −0.30, p = 0.44) and diastolic BP (r_s_ = −0.08, p = 0.84) were weak and not statistically significant. Similarly, in hypertensive participants at sea level, no significant correlations were observed for systolic (r_s_ = −0.32, p = 0.37) or diastolic BP (r_s_ = −0.22, p = 0.54).

At high altitude, normotensive participants also showed no significant associations between RMSSD and nocturnal BP (SBP: r_s_ = −0.32, p = 0.41; DBP: r_s_ = −0.13, p = 0.73). In hypertensive participants at high altitude, RMSSD displayed a moderate inverse association with nocturnal systolic BP (r_s_ = −0.45, p = 0.19) and diastolic BP (r_s_ = −0.28, p = 0.43); however, these associations did not reach statistical significance.

Overall, correlation coefficients were low across conditions, indicating that short-term cardiac vagal modulation was not consistently associated with nocturnal blood pressure variability under chronic intermittent hypoxia.

## Discussion

This study provides new evidence on nocturnal cardiovascular regulation in mine workers exposed to chronic intermittent hypoxia. Both normotensive and hypertensive workers exhibited higher nocturnal blood pressure (BP) at high altitude, accompanied by a reduction in parasympathetic-related HRV indices (RMSSD) from sea level to high altitude, and an increase in LF/HF ratio (descriptive index). The concurrent decline in RMSSD and SampEn from sea level to high altitude may indicate cardiac parasympathetic-related modulation during nighttime rest, suggesting that CIH may attenuate cardiovagal modulation during nighttime rest and be associated with a relative shift toward sympathetic predominance.

Although nocturnal BP and vagally mediated HRV indices changed in the same direction when transitioning from sea level to high altitude, their associations were weak and not statistically significant. This indicates that individual variations in cardiac autonomic modulation did not consistently parallel changes in nocturnal BP within our sample. Similar dissociations between autonomic indices and blood pressure regulation have been reported in studies of altitude-related hypoxia and hypoxic exposure (Iturriaga et al., 2014; Lang et al., 2022; Richalet et al., 2002; Farias et al., 2013; Moraga et al., 2014; Tsuji et al., 1994; Dekker et al., 1997; Roach et al., 2018; Parati et al., 2014b; Parati et al., 2025; Task Force of the European Society of Cardiology and the North American Society of Pacing and Electrophysiology, 1996; Tarvainen et al., 2014; Goldstein et al., 2011; Castiglioni et al., 2025; Prabhakar and Semenza, 2012; Fletcher et al., 1992). However, it is important to note that classical intermittent hypoxia models, characterized by short hypoxia–reoxygenation cycles lasting seconds to minutes, such as those observed in obstructive sleep apnea or experimental paradigms, differ fundamentally from occupational chronic intermittent hypoxia, which involves repeated, sustained exposures to hypobaric hypoxia over several days. Therefore, the physiological responses observed should be interpreted as the early hypoxic-phase response within a chronic intermittent exposure model, rather than as primary acute altitude exposure in unacclimatized individuals. While overlapping mechanisms may exist, including sympathetic activation, baroreflex resetting, ventilatory instability, and oxidative stress, their temporal dynamics and physiological impact are not assumed to be identical. These factors may influence blood pressure regulation through pathways that are not fully captured by short-term HRV indices.

Therefore, the absence of strong correlations in our data does not imply a lack of physiological interaction between autonomic and vascular systems. Instead, it likely reflects the multifactorial nature of BP regulation under CIH, where several pathways beyond short-term vagal modulation contribute to nocturnal BP elevation.

Although SDNN increased at high altitude, this should not be interpreted as improved autonomic or parasympathetic function. SDNN reflects total heart rate variability and includes slower oscillatory components that may be amplified under hypoxic stress. In relatively short nocturnal recordings, SDNN lacks specificity for vagal modulation (Task Force of the European Society of Cardiology and the North American Society of Pacing and Electrophysiology, 1996; Tarvainen et al., 2014; Goldstein et al., 2011; Castiglioni et al., 2025). Accordingly, RMSSD remains a more reliable marker of short-term parasympathetic-related modulation during nighttime rest.

Our findings expand previous evidence showing parasympathetic withdrawal and sympathetic predominance under CIH exposure (Lang et al., 2022), may involve mechanisms such as enhanced peripheral chemoreceptor sensitivity and altered baroreflex modulation, as suggested in previous experimental studies under hypoxic conditions (Iturriaga et al., 2009; Shi et al., 2020; Rey et al., 2008). However, these mechanisms were not directly assessed in the present study and should therefore be interpreted as physiologically plausible pathways rather than demonstrated causal mechanisms. Boos et al. (2018) reported similar reductions in HRV complexity during nocturnal high-altitude exposure (3,600 m), reinforcing the robustness of these observations in real-world settings. Despite stable resting autonomic tone at sea level, hypoxic exposure may unmask limited autonomic flexibility, particularly in individuals with underlying hypertension.

Prior research has demonstrated that CIH increases daytime and 24-h BP and promotes sympathetic excitation and vascular remodeling (Gilmartin et al., 2010; Sunderram and Androulakis, 2012). In mine workers, 24-h ambulatory BP monitoring during HA exposure consistently reveals elevated systolic and diastolic values, with the greatest increases occurring in hypertensive individuals (Lang et al., 2021). Experimental models of CIH mirror these findings, showing persistent diurnal hypertension following repeated hypoxic episodes (Sunderram and Androulakis, 2012). However, some studies suggest no long-term increase in hypertension incidence among healthy workers (Vinnikov et al., 2016), likely reflecting individual variability in CIH adaptation or baseline cardiovascular risk.

Although hematological parameters were not assessed concurrently with cardiovascular measurements, occupational records indicated values within expected ranges for workers exposed to chronic intermittent hypoxia. CIH in miners has been consistently associated with hematological acclimatization, including increased hematocrit and hemoglobin mass, reflecting hematological acclimatization to sustained hypobaric exposure. These adaptations coexist with cardiovascular changes, including right ventricular remodeling, elevated systolic blood pressure, and alterations in vascular function, although the functional implications remain heterogeneous across studies (Heinicke et al., 2003; Akunov et al., 2018; Brito et al., 2007).

Occupational roles and associated psychosocial demands may also contribute to inter-individual variability in cardiovascular and autonomic responses. Mining work involves heterogeneous workloads, prolonged 12-h shifts, night rotations, and decision-making under hypoxic conditions, all of which may increase mental workload, cognitive fatigue, and sympathetic activation (Bloch et al., 2015). These stressors, framed within the Demand–Control–Support model (Karasek, 1979), may interact with CIH exposure and influence nocturnal cardiovascular regulation. Although these factors were not specifically analyzed in the present study, all participants were exposed to comparable shift structures, and future studies should incorporate stratification by occupational role and psychosocial workload. Specifically, shift timing relative to circadian phase, prior sleep debt, and objective markers of circadian misalignment were not quantified in this protocol and may have contributed to variability in nocturnal BP and HRV responses.

Occupational factors may further interact with CIH to shape nocturnal BP regulation. Shift work, a central component of mining, has been associated with disrupted circadian rhythms, blunted dipping, and increased cardiovascular risk (Shafer et al., 2024). Early-morning or rotating shifts can induce non-dipping profiles within weeks (McHill et al., 2022). In the present study, hypertensive mine workers exhibited higher nocturnal systolic blood pressure at high altitude. Although a greater proportion displayed a non-dipping profile (<10%), between-group differences in dipping percentage did not reach statistical significance. Therefore, this observation should be interpreted as descriptive and hypothesis-generating rather than confirmatory. Non-dipping patterns have been associated with increased cardiovascular risk in other populations (Parati et al., 2014a; McHill et al., 2022) and are highlighted in current ESH guidelines (Parati et al., 2014b; Parati et al., 2025). However, the present findings do not allow definitive conclusions regarding CIH-induced alterations in dipping status. Although office blood pressure appeared controlled at sea level, ambulatory monitoring revealed altitude-related nocturnal blood pressure elevations, consistent with previous reports of masked uncontrolled hypertension under high-altitude exposure (Lang et al., 2021; Bilo et al., 2015). These findings support the potential clinical value of ambulatory and nocturnal blood pressure assessment in occupational CIH settings.

Across groups, HRV indices demonstrated weak and non-significant associations with nocturnal BP, with only weak and non-significant inverse associations between RMSSD and SBP in hypertensive workers. These findings indicate that changes in nocturnal parasympathetic modulation do not fully account for individual differences in nocturnal BP under CIH. Mechanisms such as carotid body hyperactivity, altered baroreflex function, and oxidative stress (Del Rio et al., 2010; Iturriaga et al., 2014) likely influence BP through pathways not directly captured by short-term HRV measures.

In the present cohort, four participants reported a history compatible with sleep apnea, predominantly within the normotensive group. Sleep-disordered breathing represents a relevant potential confounder in studies examining nocturnal BP and HRV, as recurrent apneic events induce intermittent hypoxemia, sympathetic surges, blood pressure elevations, and alterations in cardiac autonomic modulation (Moraga et al., 2014; Sforza and Roche, 2016; Parati et al., 2019). These pathophysiological mechanisms partially overlap with those observed in chronic intermittent hypoxia, although their temporal patterns differ substantially.

Importantly, continuous nocturnal oxygen saturation and respiratory event monitoring were not performed in this field-based protocol. Therefore, we cannot exclude the presence of unrecognized obstructive or central apnea episodes, particularly during the first night at high altitude, when ventilatory instability and periodic breathing may be accentuated. Hypobaric hypoxia may exacerbate both central apneas and oxygen desaturation episodes, which in turn can influence nocturnal BP regulation independently of baseline autonomic tone.

Consequently, the observed elevations in nocturnal BP and reductions in vagal-related HRV indices may reflect a combined effect of sustained hypobaric hypoxia and potential sleep-disordered breathing phenomena. Future studies incorporating polysomnography or continuous nocturnal oximetry are warranted to disentangle these overlapping mechanisms under occupational CIH exposure.

From a clinical perspective, the coexistence of nocturnal hypertension and reduced parasympathetic modulation highlights a physiologically vulnerable pattern in high-altitude mine workers. Because HRV and BP provide complementary information on cardiovascular regulation, nocturnal assessment of both parameters, particularly during the first nights at altitude, may contribute to improved physiological monitoring frameworks in occupational CIH settings; however, outcome validation studies are required before formal surveillance recommendations can be established. A key strength of this study is the synchronized nocturnal monitoring of HRV and ambulatory BP under real occupational CIH exposure, using validated devices and a standardized protocol. Inclusion of participants with ≥4 h of artifact-free ECG data ensured high-quality nocturnal autonomic assessment. Furthermore, comparing normotensive and hypertensive workers across controlled SL and HA conditions enhances the clinical relevance of our findings.

### Limitations and strengths

This study was conducted under real-world operational conditions in rotating-shift mining workers exposed to chronic intermittent hypoxia and should be interpreted as a pilot field-based investigation. The logistical constraints inherent to active mining environments precluded comprehensive sleep assessment (e.g., polysomnography or EEG) and continuous nocturnal oxygen saturation monitoring. Therefore, the present findings reflect cardiovascular and autonomic behavior during a standardized nighttime rest window rather than sleep-stage–specific physiology.

Several methodological constraints in this study are structural and should be considered central to the interpretation of the findings, particularly the male-only sample, the use of a standardized 4-h nighttime window rather than full polysomnographic sleep assessment, and the reliance on single-lead ECG recordings for HRV analysis.

The absence of detailed sleep architecture and direct respiratory measurements limits stage-specific interpretation. Respiratory rate was estimated using ECG-derived respiration, which represents an indirect surrogate. Non-linear HRV indices, including sample entropy, may be sensitive to signal length and sleep-stage composition; although a standardized 4-h window and uniform preprocessing were applied across conditions, these measures should be interpreted cautiously.

The relatively small sample size, particularly after stratification, limits statistical power and may increase susceptibility to both type I and type II errors when evaluating multiple HRV indices. RMSSD was defined *a priori* as the primary parasympathetic-related marker, whereas additional HRV metrics were considered secondary and descriptive. Antihypertensive therapy within the hypertensive subgroup was heterogeneous; although medication remained stable across conditions and no participants were treated with beta-blockers, class-specific pharmacological effects cannot be entirely excluded. Differences in occupational roles and daily workload were not formally quantified and may have contributed to inter-individual variability.

Despite these limitations, the major strength of this study lies in its execution under real working conditions in an active high-altitude mining operation. Unlike laboratory-based or simulated hypoxia studies, these data reflect the true physiological demands faced by workers during routine high-altitude shifts and provide clinically and occupationally relevant insights to guide future mechanistic investigations.

Accordingly, the present results describe physiological patterns observed under operational CIH exposure rather than definitive mechanistic or stage-specific sleep conclusions.

## Conclusion

This study describes nocturnal cardiovascular and autonomic patterns observed in male high-altitude mine workers exposed to chronic intermittent hypoxia under real-world operational conditions. CIH exposure was associated with higher nocturnal blood pressure and reduced parasympathetic-related HRV indices during a standardized nighttime rest period, with more pronounced nocturnal BP increases observed in hypertensive participants. These findings describe cardiovascular and autonomic patterns observed during the early hypoxic phase of a long-standing rotational CIH exposure model.

Although autonomic and hemodynamic changes occurred in parallel at the group level, their individual associations were weak and inconsistent. These findings suggest that short-term cardiac autonomic modulation alone may not fully explain nocturnal blood pressure responses under hypoxic stress.

Given the restricted nocturnal sampling window and the demographic characteristics of the cohort, the present results should be interpreted as field-based physiological observations rather than definitive mechanistic conclusions. Further studies incorporating full-night recordings, sleep-stage characterization, and more diverse populations are warranted to refine understanding of cardiovascular regulation under occupational CIH exposure.

## Data Availability

The datasets presented in this article are not readily available because the dataset contains sensitive physiological and occupational information from a small group of mining workers. Due to ethical restrictions, confidentiality requirements, and agreements with the mining company, the raw data cannot be shared. Requests to access the datasets should be directed to morin.lang@uchile.cl.
