# Supplementary material for: Cardiovascular and autonomic modulation during nighttime rest under real-world conditions in miners exposed to chronic intermittent hypoxia
Source: Front Physiol. 2026 Mar 30;17:1747092. doi: 10.3389/fphys.2026.1747092 (PMC13070778; doi:10.3389/fphys.2026.1747092)
Supplement: Supplementary file 1 [file DataSheet2.pdf]

Supplementary Material

Supplementary Figures and Tables

This supplementary file provides additional data supporting the main findings related to cardiovascular, respiratory, and symptom responses under repeatedly chronic intermittent hypoxia (CIH).

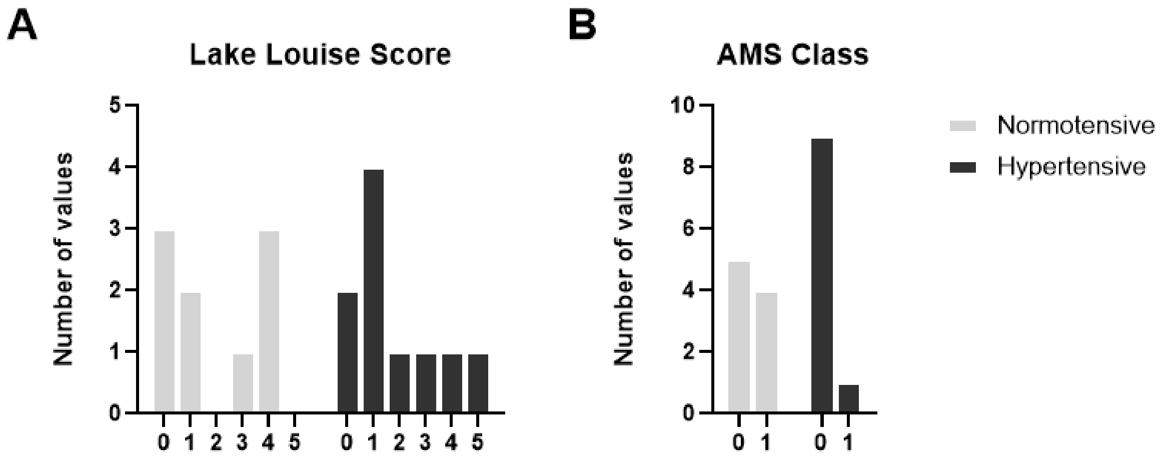

Supplementary Figure 1. Distribution of Lake Louise Scores and acute mountain sickness (AMS) classification among miners during the first night at high altitude.

(A) Frequency distribution of LLS in normotensive (grey bars) and hypertensive (black bars) participants.
(B) Classification of participants into AMS Class 0 (no AMS) or Class 1 (AMS present) based on Lake Louise criteria. **Abbreviations:** LLS, Lake Louise Score; AMS, acute mountain sickness.

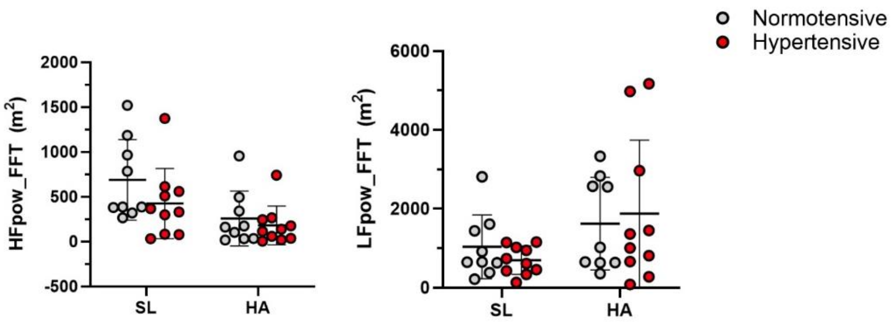

Supplementary Figure 2. Absolute spectral power (LF and HF, ms²) during the standardized nocturnal window (00:30–04:30 h), stratified by blood pressure status. Individual values and group mean ± SD are shown. Normotensive participants are shown in white and hypertensive participants in red. Asterisks indicate within-group comparisons between SL and HA (Wilcoxon signed-rank test). No significant between-group differences were observed at either altitude.

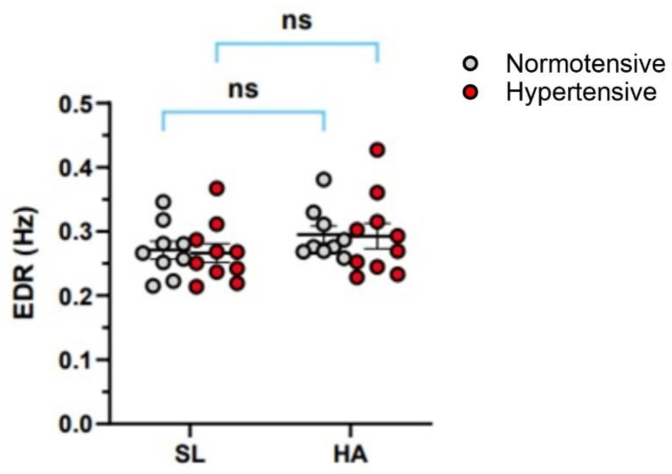

Supplementary Figure 3. ECG-derived respiratory rate (EDR, breaths·min^{-1}) during the 4-hour nocturnal window at sea level (SL) and high altitude (HA). Individual values for each participant are shown for normotensive (white) and hypertensive (red) miners at both altitudes during the standardized nocturnal period (00:30–04:30). Horizontal lines represent group means. No significant differences were observed between altitudes or clinical groups. **Abbreviations:** EDR, ECG-derived respiration; SL, sea level; HA, high altitude
