# Supplementary figures and images for: Cardiovascular and autonomic modulation during nighttime rest under real-world conditions in miners exposed to chronic intermittent hypoxia
Source: Front Physiol. 2026 Mar 30;17:1747092. doi: 10.3389/fphys.2026.1747092 (PMC13070778; doi:10.3389/fphys.2026.1747092)

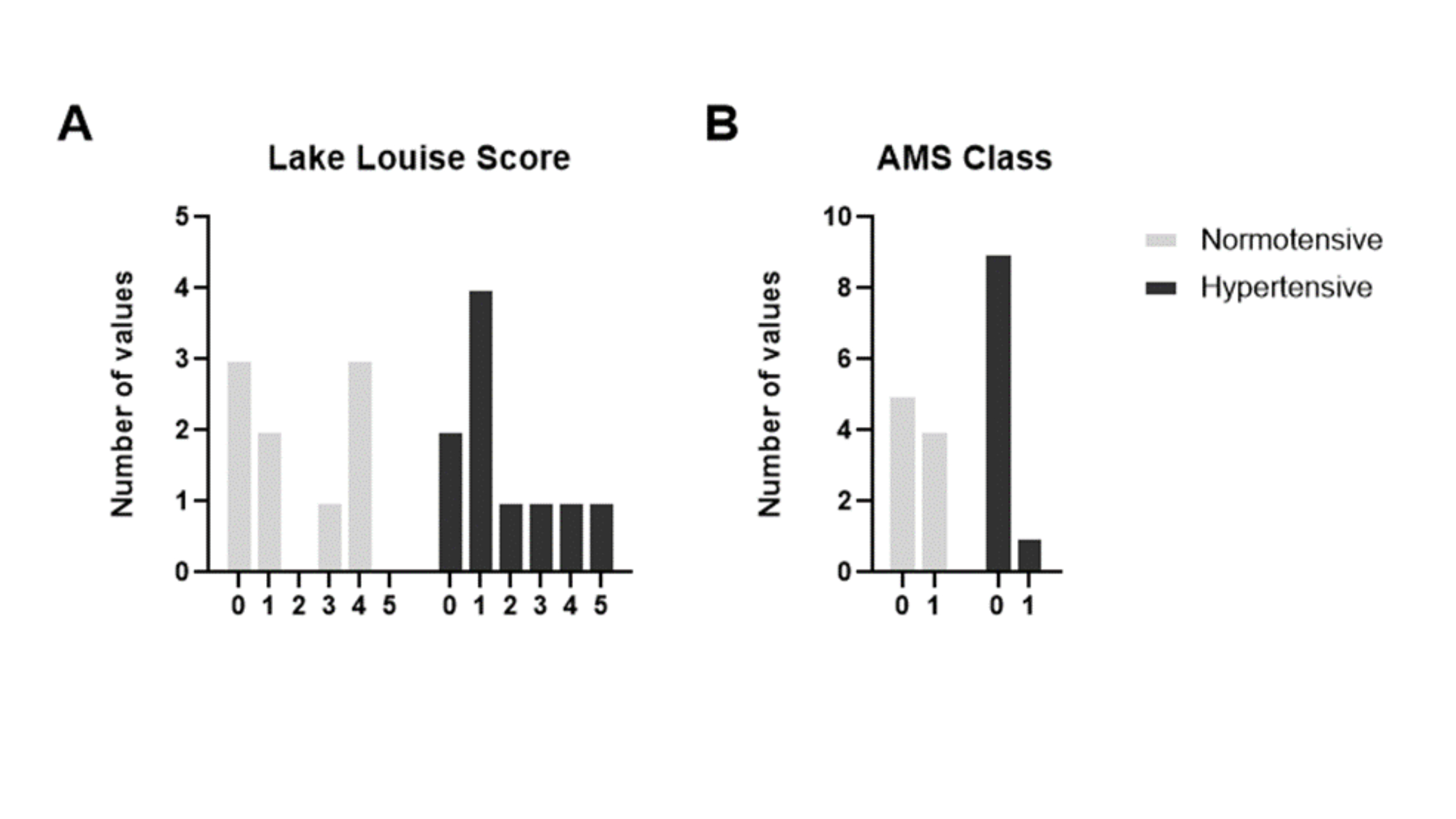

Supplement: Supplementary file 2 [file Image3.jpeg]

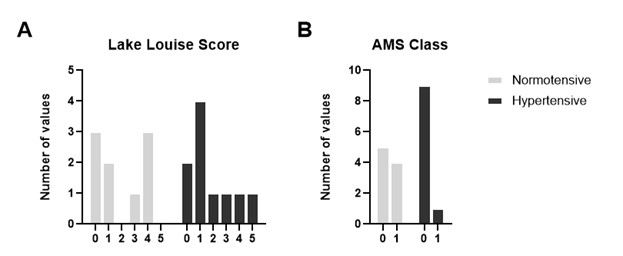

Supplement: Supplementary file 3 [file Image1.jpeg]

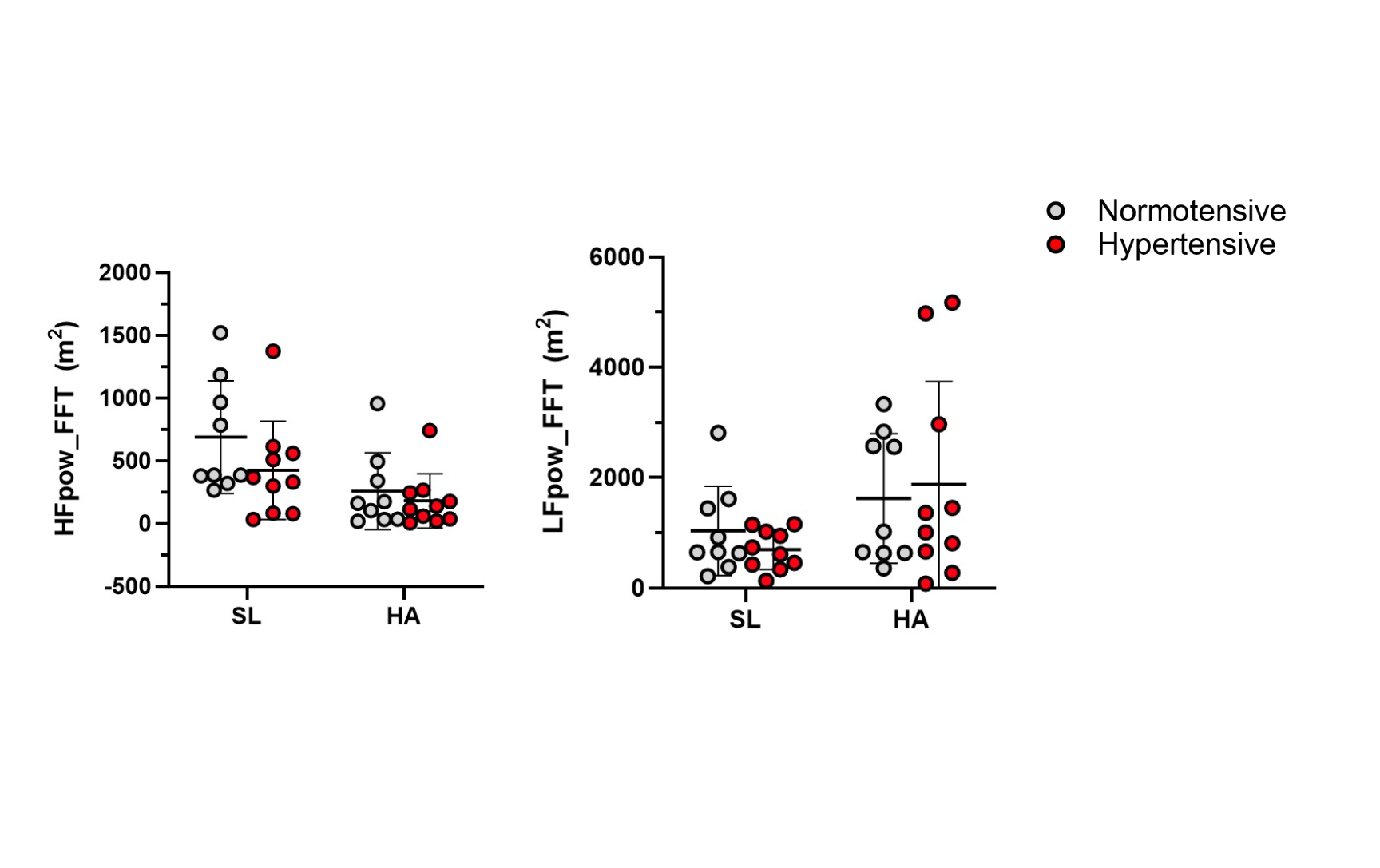

Supplement: Supplementary file 4 [file Image4.jpeg]

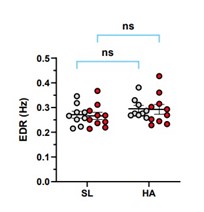

Supplement: Supplementary file 5 [file Image2.jpeg]

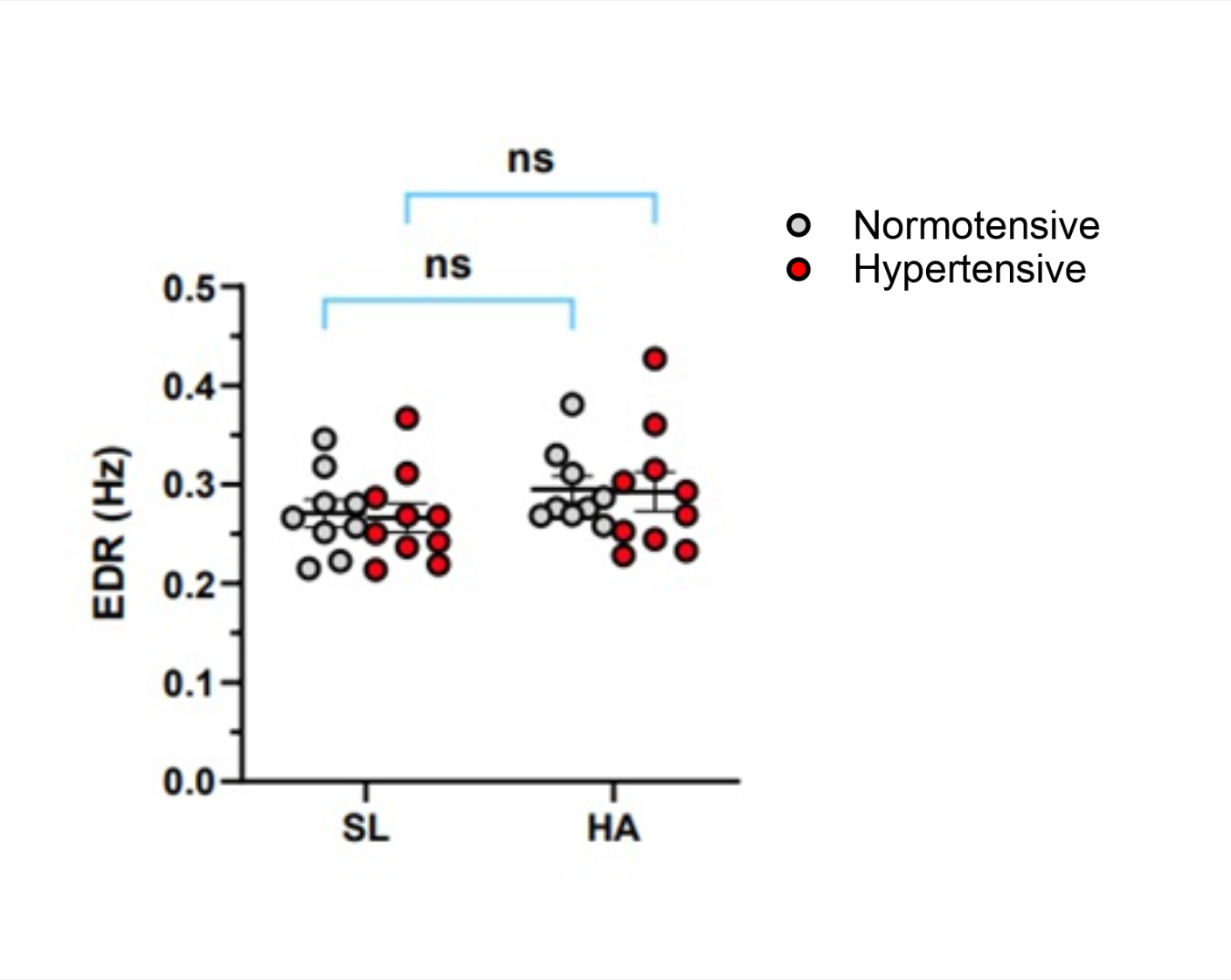

Supplement: Supplementary file 6 [file Image5.jpeg]
